# A complicated path to the CRMO diagnosis – case of a 9 year old girl whose story comes full circle

**DOI:** 10.1186/s12891-019-2776-9

**Published:** 2019-08-31

**Authors:** Joanna Świdrowska-Jaros, Elżbieta Smolewska

**Affiliations:** Department of Paediatric Cardiology and Rheumatology, Medical University of Lodz, Łódz, Poland

## Abstract

**Background:**

Chronic recurrent multifocal osteomyelitis (CRMO) is a rare idiopathic autoinflammatory bone disease that mostly affects children and adolescents. It is a diagnosis of exclusions since no clinical signs and symptoms are pathognomonic. Radiological tests are often essential, but bone biopsy may be needed in unclear cases.

**Case presentation:**

A 9-year-old Caucasian girl with a history of bone pain. The data from the history and results of laboratory tests suggested osteomyelitis, but no adequate response to the treatment was observed. A number of imaging tests did not confirm the diagnosis, therefore a bone biopsy was necessary.

**Conclusions:**

Differential diagnosis of CRMO is challenging and it is based on exclusions. Since it might be misdiagnosed or mistreated, bone biopsy should be considered in patients reporting bone pain who are unresponsive to treatment.

## Background

Chronic recurrent multifocal osteomyelitis (CRMO) is an autoinflammatory bone disease of unknown origin, that mostly affects children and adolescents [1]. Symptoms of CRMO might be varied - from asymptomatic single-bone involvement to chronic, recurrent, multifocal inflammation with systemic symptoms such as weakness, febrile states and weight loss [2]. Bone lesions are located in various skeletal sites, mainly in long bones metaphyses (tibia, thigh, arrow), pelvic bones, spine, clavicle or mandible [3]. A diagnostic path is complicated and includes laboratory tests and variation of imaging examinations. Routine inflammation markers are often in a normal range and the imagining may result unclear – in that cases, bone biopsy might be needed. Differential diagnosis is difficult and contains malignancies, chronic infections or other systemic diseases [4, 5].

## Case presentation

A 9-year-old Caucasian girl was referred to the Department of Paediatric Cardiology and Rheumatology of Medical University of Lodz with a history of bone pain for the last one month. The pain was localized in the lower extremities, waking up at night and making it difficult to walk. Any peripheral joint oedema had been observed. The patient was otherwise healthy, no fevers, chills or weight loss were reported. She had no family history of bone or joint abnormalities, including tumors. She had no history of trauma or any triggering factor for the onset of the pain.

At the time of initial evaluation, she was reporting pain throughout the day and night. Pain was quantified as 8 out of 10 in the visual analogue scale (VAS). The left ankle was slightly swollen and was tender to touch, but no increase of the local temperature was observed. The active and passive motion ranges of the lower extremity joints – such as hips, knees and ankles - were decreased, due to severe pain. In otherwise general physical exam including skin and neurological exam was normal, no infection foci were found elsewhere in the body.

Her initial blood work revealed high rates of inflammation markers (CRP 119,1 mg/l *N* < 5,0; ESR 135 mm/h) with normal hematological and biochemical parameters (including lactate dehydrogenase, alkaline phosphatase or uric acid). At the day of admission, ultrasound examination of peripheral joints of the lower limbs was performed, no joint effusion and any other joint inflammation features were found. Empirical antibiotic (ceftriaxone) therapy together with non-steroidal anti-inflammatory drugs was implemented, however, the inflammatory markers’ levels were worsening in the control laboratory tests. Antibiotic treatment was extended with aminoglycoside, the girl reported aggravating of the night pain, migrating from bone to bone, night sweats were additionally observed. Blood culture was negative, but serologic tests had detected positive antibody titers to various pathogens (EBV, Yersinia spp., Mycoplasma and Chlamydia pneumoniae, Salmonella spp., Borrelia burgdorferi) - that was probably associated with immunological disorders.

**Fig. 1:**
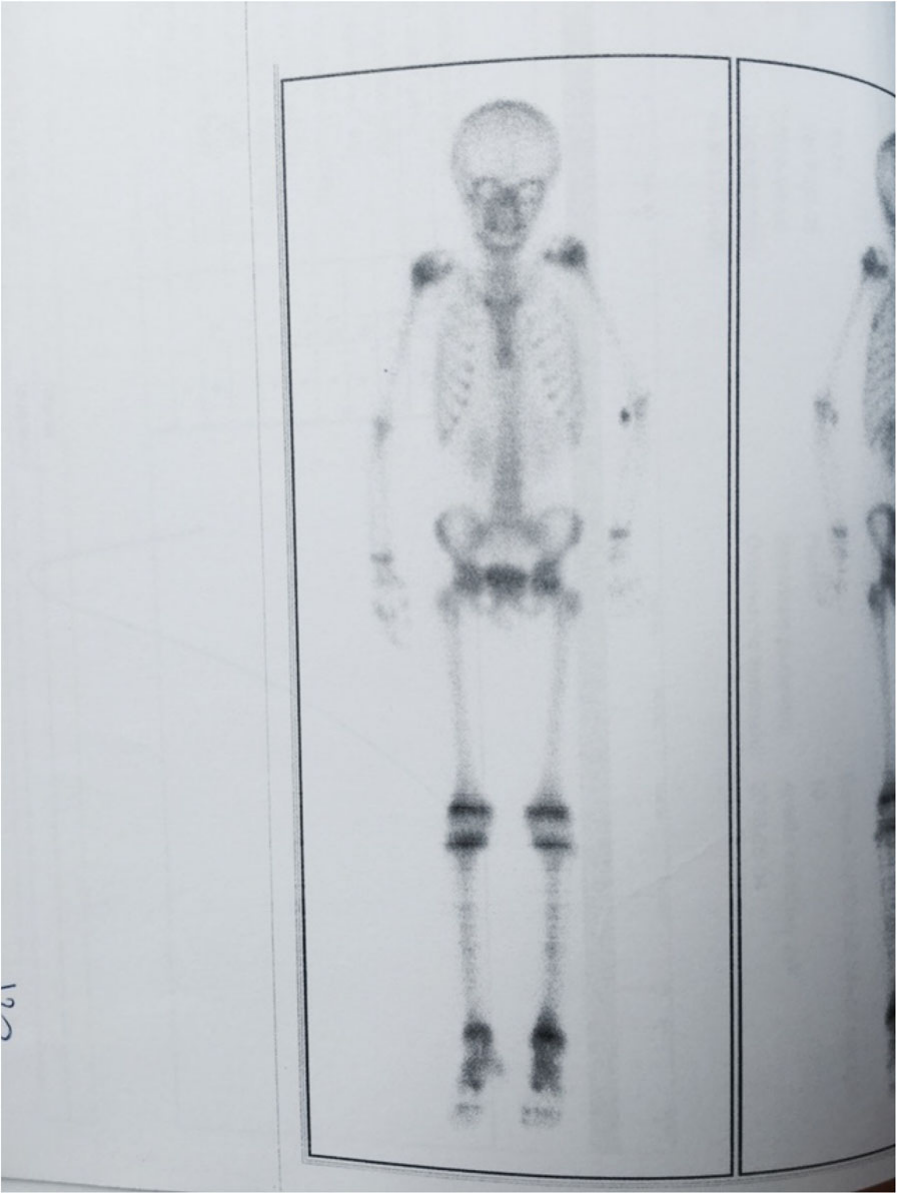
Isotope bone scan revealing increased uptake in the femur, humerus and tibia

**Fig. 2:**
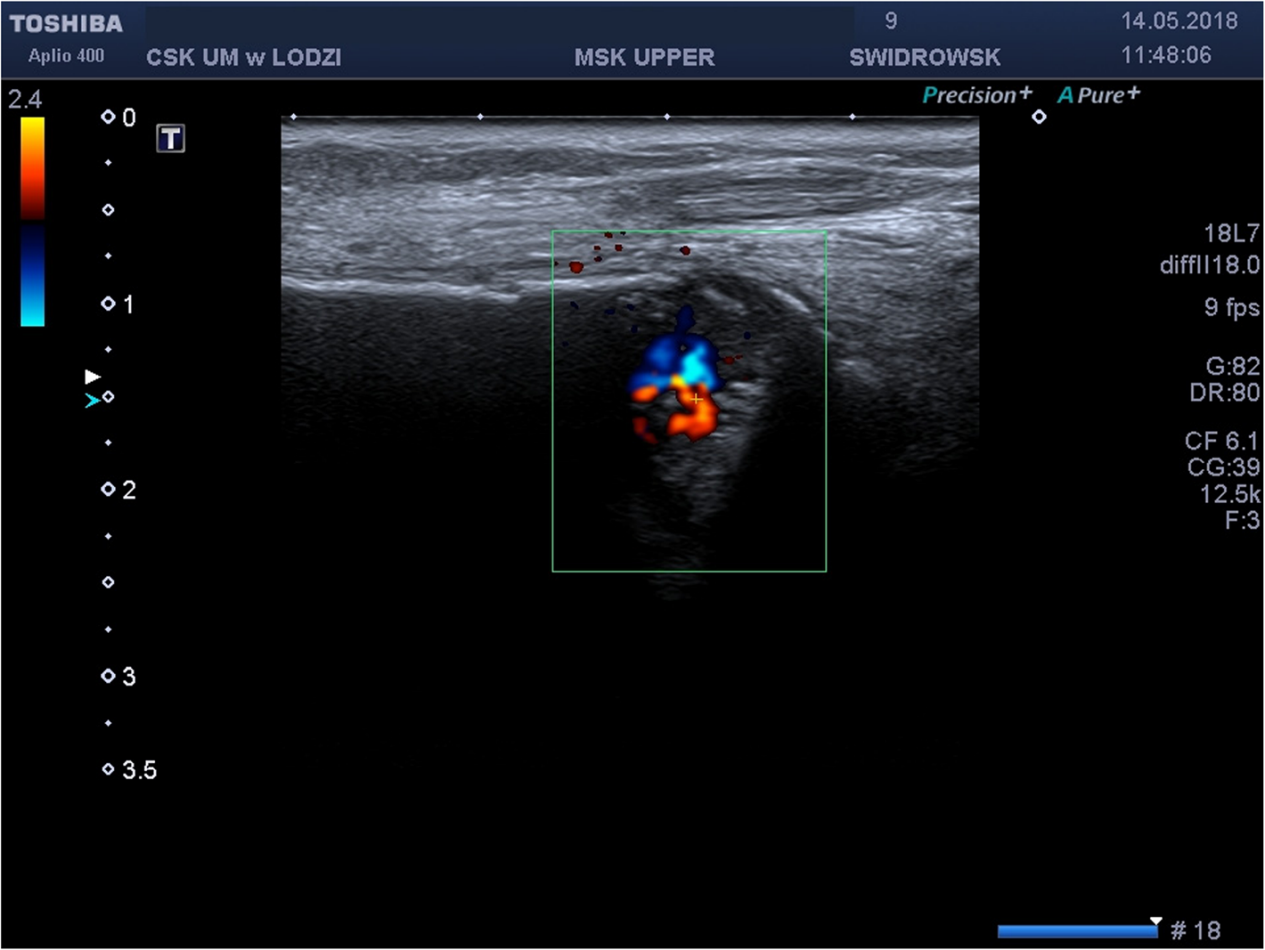
Ultrasound examination of an ankle area showing destruction of distal metaphysis of the left tibia

**Fig. 3:**
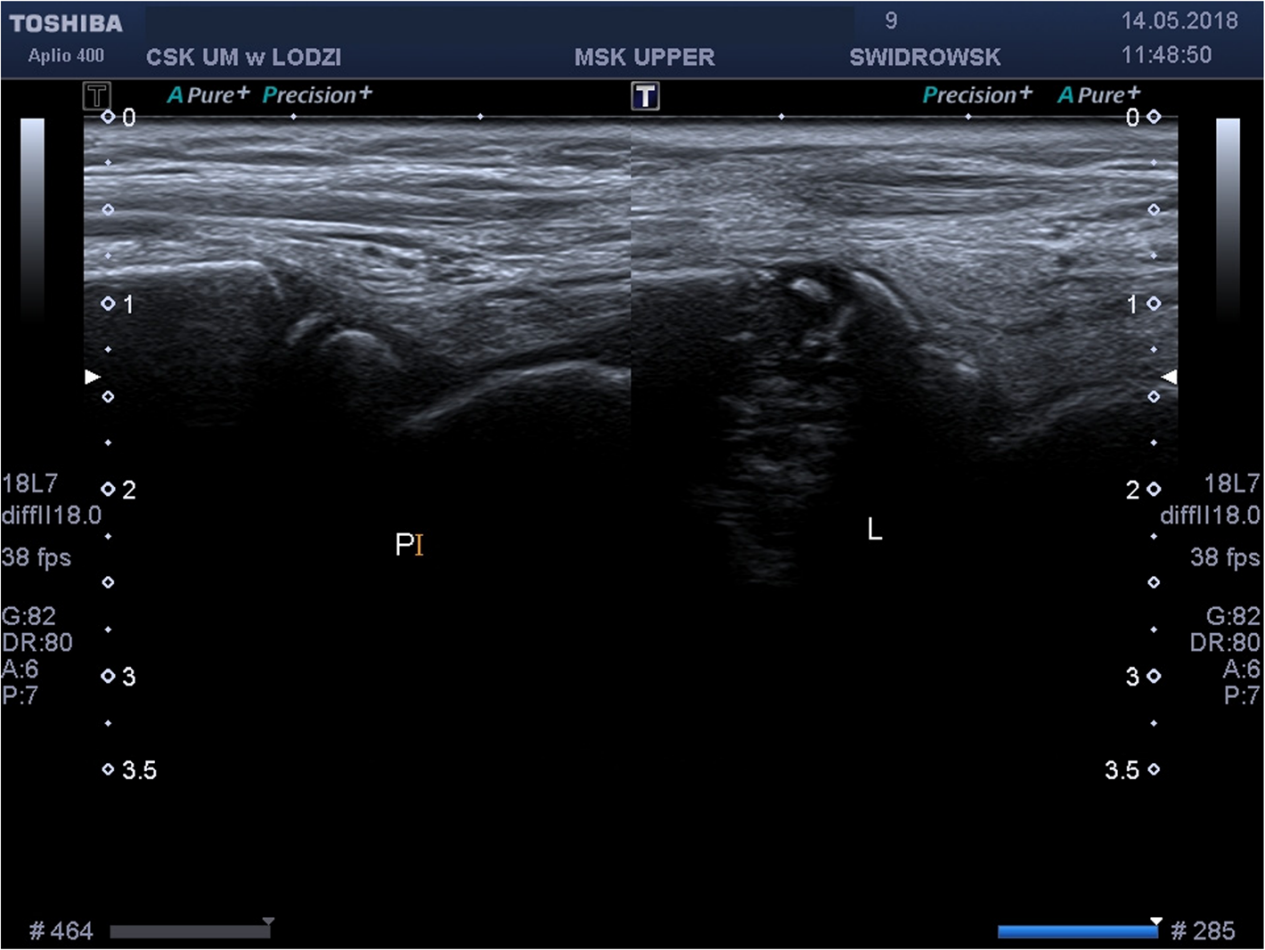
Ultrasound examination of an ankle area showing destruction of distal metaphysis of the left Tibia

**Fig. 4:**
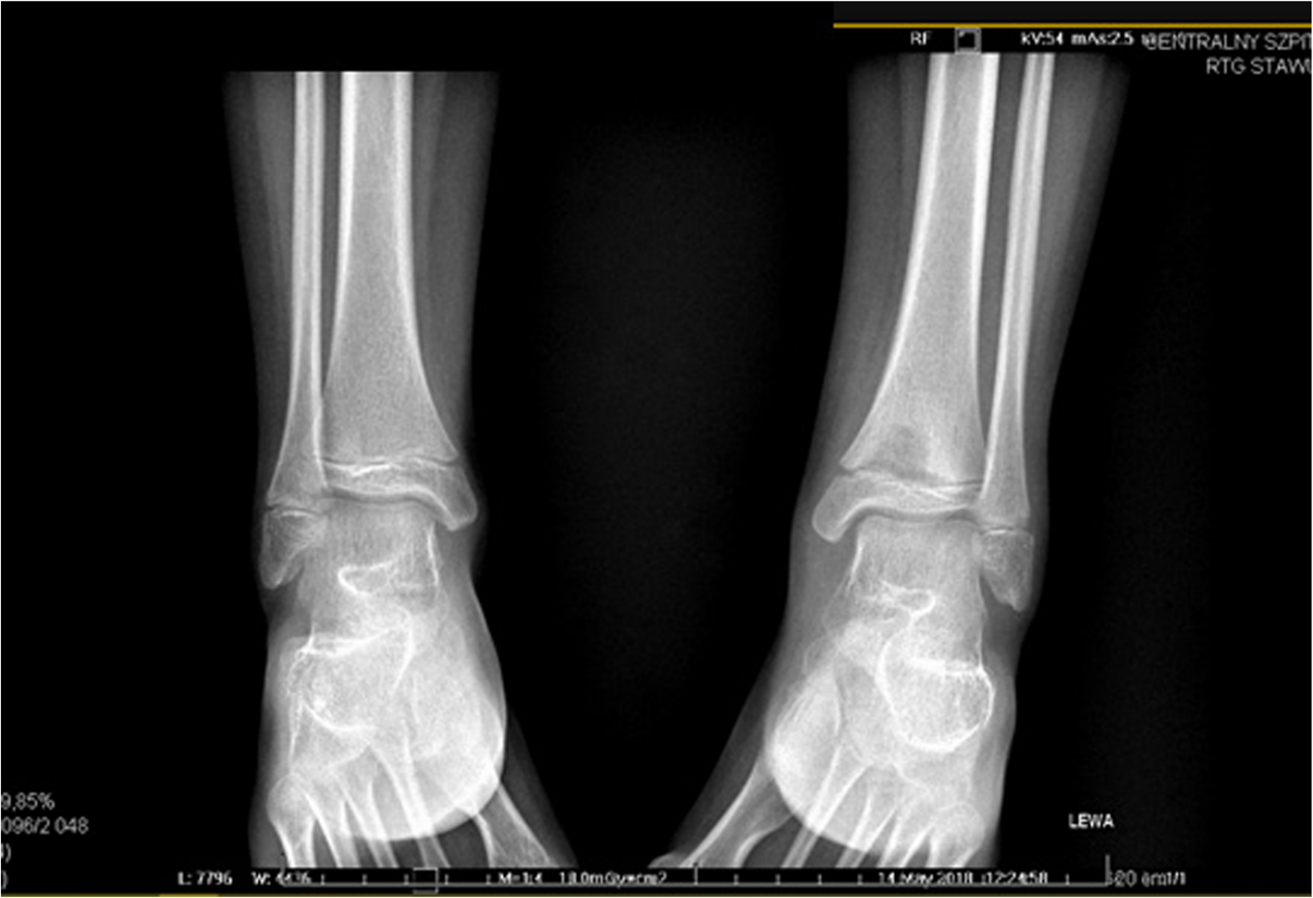
Radiograph showing a large lytic lesion in the metaphysis of left tibia

**Fig. 5:**
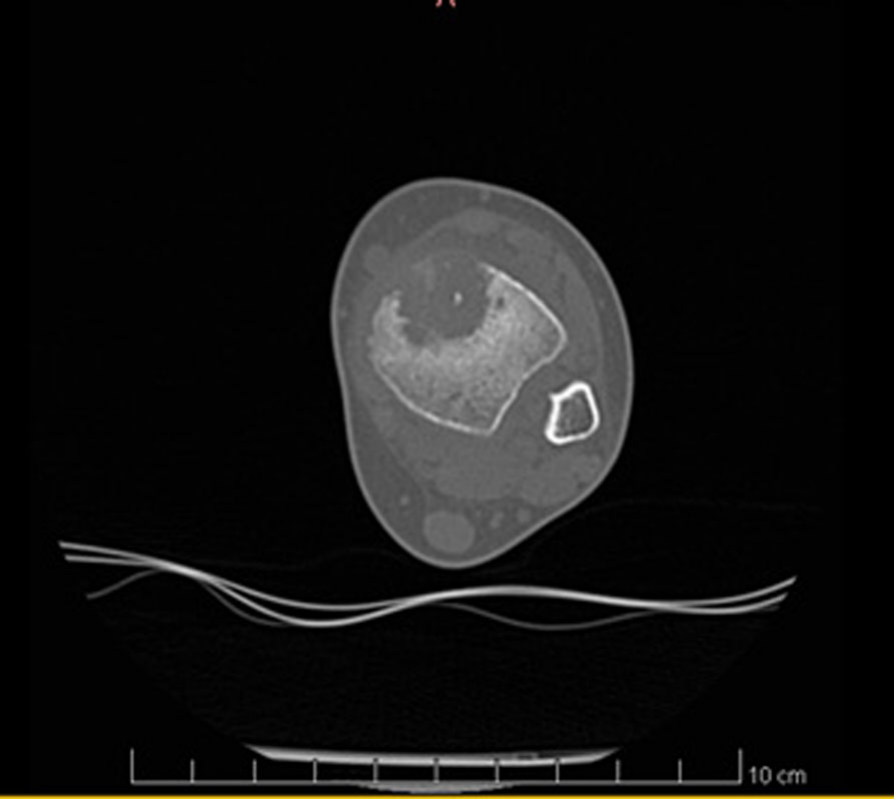
CT scan showing osteolytic lesion of the metaphysis of left tibia

**Fig. 6:**
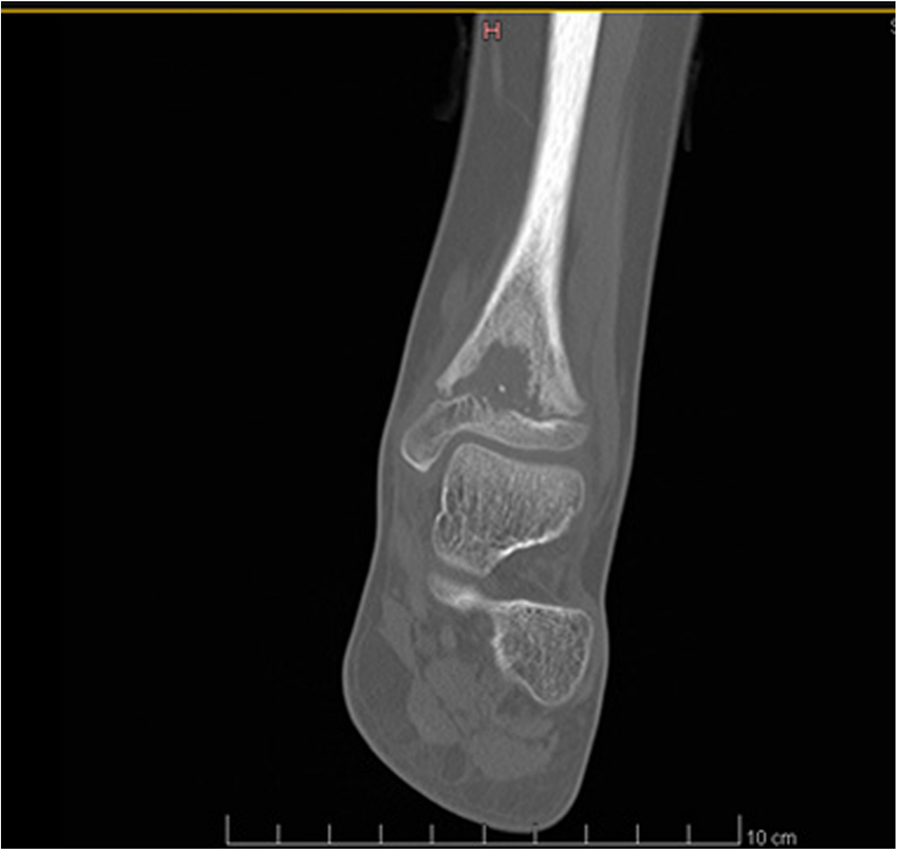
CT scan showing osteolytic lesion of the metaphysis of left tibia

**Fig. 7:**
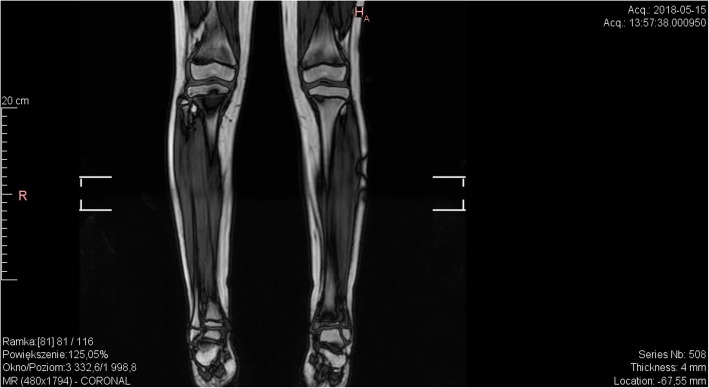
Whole body NMR revealing multiple foci of elevated marrow signal

**Fig. 8:**
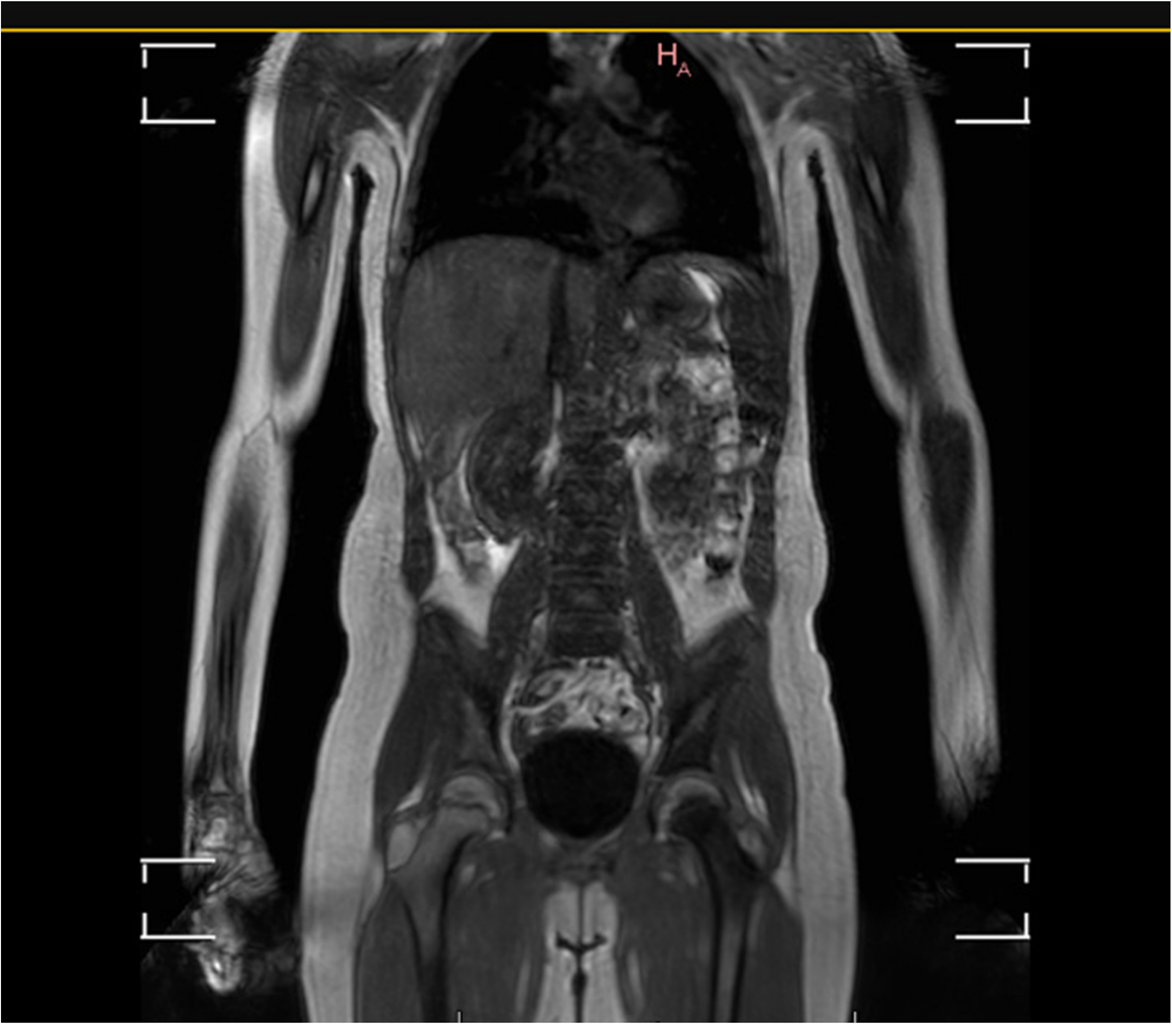
Whole body NMR revealing multiple foci of elevated marrow signal

**Fig. 9:**
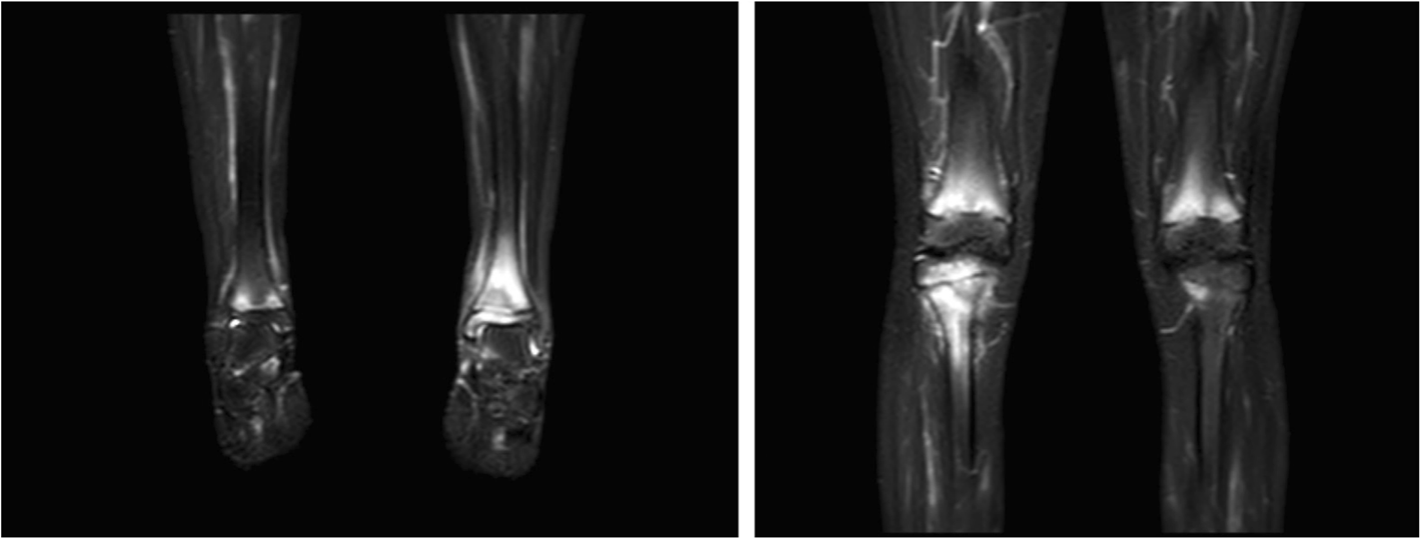
STIR fa suppression image of lesions in proximal and distal metaphysis of left tibia

It was decided to perform isotope bone scan (Fig. 1) revealing increased uptake in the femur, humerus and tibia, that could correspond to bone inflammation. Despite prolonged antibiotic treatment, no therapeutic effect was achieved - inflammatory parameters were increasing, gradual anemization appeared. Ultrasound examination of peripheral joints was made again (Figs. 2, 3), showing a large progression of lesions. Destruction of distal metaphysis of the left tibia and the outline of the left femoral head was found with the inflammation features penetrating throughout the bone. A suspicion of proliferation process was made based on the radiograph (Fig. 4) and a computed tomography of the ankle area (Figs. 5, 6). It was decided to perform the bone marrow biopsy, which showed no significant abnormalities. No non-specific tumor markers (NSE, AFP, beta-HCG and chromogranin A) were found positive. NMR-DWIBS examination (Figs. 7, 8 and 9) revealed numerous osteolytic foci suggesting a disseminated tumor process - with no apparent pathological mass in both the abdominal cavity and the chest. The decision was made to transfer the patient to the Department of Pediatric Oncology and Oncologic Surgery of Institute of Mother and Child in Warsaw, where the antibiotic treatment had been suspended and bone biopsy was performed. Since the malignancy was ruled out and no bacterial culture were positive, the diagnosis of CRMO was considered at this point. The girl was referred back to our Department, where the antibiotic treatment was stopped as ineffective, and non-steroidal anti-inflammatory medication (naproxen) was again introduced. Due to the unsatisfactory control of the inflammatory process, systemic steroids and sodium pamidronate infusions were used, resulting in significant clinical improvement, relief of pain and decrease in the value of inflammation markers. NMR-DWIBS was performed after two cycles of treatment with bisphosphonates and revealed a large regression of osteolytic lesions including the major focus in the metaphysis of left tibia. No new osteolysis foci were observed.

## Discussion

Diagnostic path of CRMO is complicated due to the overlap of clinical and imaging findings [6]. It often remains a diagnosis of exclusions between tumors and infectious arthritis [7]. Unfortunately, there are various criteria according to different authors. The latest criteria belongs to *Roderick* et al (Bristol diagnostic criteria for CRMO). Authors suggest, that CRMO might be suspected in case of a bone pain with or without swelling and without significant features of infection, with the typical radiological findings (lytic areas, sclerosis, new bone formation) and – if the disease is multifocal, with no CRP level elevation, but - if the disease affects one bone – with CRP level greater than 30 g/l and the bone biopsy showing inflammatory changes with no bacterial growth while not on antibiotic therapy [8]. *Manson* et al proposed the criteria that includes remission and exacerbation of signs and symptoms for at least 6 months, lack of an identifiable cause, lack of response to antibiotics for at least one month and the chronic, nonspecific inflammation consisting of lymphocytes, plasma cells and histiocytes at histopathologic examination [9]. Otherwise, *Beretta-Piccoli* et al advise that CRMO can be diagnosed if the disease course lasts at least 3 months in duration, there is a bioptical evidence of chronic bone inflammation with the exclusion of other diseases, and there is a failure to cultivate an organism [10].

Exclusion of other etiologies are the main purpose on the diagnosis of CRMO, since no specific diagnostic biomarkers are available [11]. Malignancies are the first to rule out by the clinician - Ewing’s sarcoma, osteosarcoma, Langerhans cell histiocytosis [12–15]. Multifocal involvement is helpful in establishing the diagnosis, however – when there is a single or atypical lesion, the diagnostic pathway might be challenging.

Routine inflammation markers are in a normal range in the majority of affected subjects. However, *Brown* et al and *Catalano-pons* et al reported inflammatory markers increased in more than a half of the examined patients [16]. In our case - highly elevated CRP and ESR got the clinician confused and suggested infectious osteomyelitis. Since there was no response to antibiotic treatment, further investigation was performed. Imaging in CRMO is not clear though. Having a multifocal localization - radioisotope bone scan may be a useful tool in establishing the diagnosis and identifying clinically silent lesions that are may be present at the initial stage [17, 18]. Radiographic evaluation can be characteristic but not pathognomonic, although computed tomography has a limited role in the diagnosis of CRMO [7, 15]. Both methods work parallel and might demonstrate decalcification or osteolysis, periosteal reaction and the bone destruction. Initial radiographs show metaphyseal disease (lytic lesions adjacent to the growth plate). MRI can be useful to clearly characterize the type of lesions and may help in determining the best biopsy location [19]. Moreover, the whole body MRI could assist in evaluating and excluding a pathological mass wherever in the body [20, 21]. The proper image strategy, suggested by *Handrick* et al, should be as follows: radiographs, bone scintigraphy, MR imaging [22]. In case of our patient, all of the imaging methods mentioned above were used, but no diagnosis was set. To rule out both - chronic bacterial osteomyelitis and malignancy - definite diagnosis relies on histopathological confirmation done by bone biopsy.

Our patient fulfills most of the clinical features that are present in other previous reports. CRMO occurs mainly in children and adolescents and affects the girls aged to be about 10. The most often affected location are the lower extremity bones. Initial symptoms including bone pain are reported in the majority of patients [8, 23, 24].

As our patient presented multiple bone lesions, had a biopsy indicative of chronic inflammation and a negative blood culture, the diagnosis of CRMO was confirmed.

Treatment guidelines for CRMO are still under discussion and include watchful waiting for spontaneous remission, although the therapy of first choice consist of nonsteroidal anti-inflammatory drugs [28]. Glucocorticosteroids, bisphosphonates including pamidronate, sulfasalazine, anti-tumor necrosis factor α (TNFα) drugs have been used with good results (Table 1) [25–27]. Antibiotic treatment is considered ineffective [2, 6]. In our case, the patient underwent pharmacological therapy with glucocorticosteroids, sulfasalazine and pamidronate and responded well to introduced treatment. After two courses of bisphosphonates therapy - went to remission, no pain was reported, inflammation markers decreased.

**Table 1:** Treatment protocol for CRMO

First line treatment – NSAIDs:	
Ibuprofen 30–40 mg/kg/day in 3–4 divided doses for 1–3 months Naproxen 10-15 mg/kg/day in 2 divided doses for 1–3 months	
Second line treatment – Corticosteroids:	
Prednisolone 1–2 mg/kg/day in 1 dose for 5–10 days up to clinical improvement, NSAIDs as the continuation of the therapy; In severe cases prednisolone treatment may be prolonged up to 4–6 weeks	
Third line treatment – DMARDs	
Sulfasalazine Methotrexate Bisphosphonates (Pamidronate) TNFα-inhibitors	

## Conclusion

Differential diagnosis of CRMO is challenging and it is based on exclusions. Since it might be misdiagnosed or mistreated, bone biopsy should be considered in patients reporting bone pain who are unresponsive to treatment.

## Data Availability

Available in the Department of Pediatric Cardiology and Rhematology, Medical Univrsity of Lodz, Poland.

## Abbreviations

AFP: Alpha fetoprotein
CRMO: Chronic recurrent multifocal osteomyelitis
CRP: C-reactive protein
DWIPS: Diffusion-weighted whole-body imaging with background body signal suppression
EBV: Epstein-Barr Virus
ESR: Erythrocyte sedimentation rate
NMR: Nuclear magnetic resonance
NSE: Neuron-specific enolase
TNF: Tumor necrosis factor
VAS: Visual analogue scale
